# Improving pathways to primary health care among LGBTQ populations and health care providers: key findings from Nova Scotia, Canada

**DOI:** 10.1186/s12939-018-0786-0

**Published:** 2018-06-13

**Authors:** Jacqueline Gahagan, Montse Subirana-Malaret

**Affiliations:** 1 School of Health & Human Performance, Health Promotion, Gender & Health Promotion Studies Unit (GAHPS Unit), Healthy Populations Institute (HPI), Dalhousie University, 6230 South Street, Halifax, NS B3H 3J5 Canada; 2 Advanced Studies Group on Violence, Clinical Psychology and Psychobiology Unit, Universitat de Barcelona, Faculty of Psychology, Passeig de la Vall d’Hebron, 171, 08035 Barcelona, Spain

**Keywords:** LGBTQ, Primary Health, Health providers, Health system, Nova Scotia

## Abstract

**Background:**

This study explores the perceived barriers to primary health care as identified among a sample of Lesbian, Gay, Bisexual, Transgender, and Queer (LGBTQ) identified individuals and health care providers in Nova Scotia, Canada. These findings, based on a province-wide anonymous online survey, suggest that additional efforts are needed to improve pathways to primary health among LGBTQ populations and in deepening our understanding of how to advance the unique primary health needs of these populations.

**Methods:**

Data were collected from the LGBTQ community through an online, closed-ended anonymous survey. Inclusion criteria for participation were self-identifying as LGBTQ, offering primary health care to LGBTQ patients, being able to understand English, being 16 years of age or older, and having lived in Nova Scotia for at least one year. A total of 283 LGBTQ respondents completed the online survey which included sociodemographic questions, perceptions of respondents’ health status, and their primary health care experiences. In addition, a total of 109 health care providers completed the survey based on their experiences providing care in Nova Scotia, and in particular, their experiences and perceptions regarding LGBTQ access to primary health care and physician-patient interactions.

**Results:**

Our results indicate that, in several key areas, the primary health care needs of LGBTQ populations in Nova Scotia are not being met and this may in turn contribute to their poor health outcomes across the life course.

**Conclusion:**

A framework of intersectionality and health equity was used to interpret and analyze the survey data. The key findings indicate the need to continue improving pathways to primary health care among LGBTQ populations, specifically in relation to additional training and related supports for health care providers who work with these populations.

## Background

According to the WHO [1], primary health includes five key elements which are related to “reducing exclusion and social disparities in health (universal coverage reforms); organizing health services around people’s needs and expectations (service delivery reforms); integrating health into all sectors (public policy reforms); pursuing collaborative models of policy dialogue (leadership reforms); and increasing stakeholder participation”. However, the issues of exclusion and disparities in relation to primary health can result in certain populations not availing themselves of health care [2–5]. This is particularly the case for Lesbian, Gay, Bisexual, Transgender and Queer (LGBTQ) populations whose unique health needs may not be considered in primary health where ‘health’ has been constituted historically through a cisnormative and heteronormative framework [6–8]. Moreover, this can impact on optimal health and well-being across the life course, particularly given that LGBTQ populations are less likely to access primary health care services for fear of discrimination and stigma [4, 9, 10]. In addition, recent research has shown that many practicing health care professionals and health care trainees are often lacking knowledge, comfort or cultural competence in addressing a variety of health issues facing LGBTQ populations [11–14]. Cultural competence is an important consideration in primary health care as it refers to a set of attitudes, behaviours and policies to work effectively in cross-cultural settings [15]. A lack of access to culturally competent primary health care systems and providers can result in increased costs to society, including reduced life expectancy, a lower quality of life, and a higher burden of acute and chronic illness among LGBTQ populations (e.g. cancer, HIV, CVD) [16–18]. Previous research has also attributed higher rates of chronic disease among LGBTQ populations to discrimination, minority stress, avoidance of health care providers and irregular access to health care services [19, 20]. Therefore, developing culturally competent primary health care approaches for LGBTQ patients has become a priority in many health research and clinical practice settings [4, 21]. Given this, our study explores the main health concerns and perceived pathways and barriers to primary health care for LGBTQ populations from the perspective of both a sample of LGBTQ community members and health care providers.

## Methods

### Respondents

Data were collected from LGBTQ communities in Nova Scotia, Canada, one of the largest provinces on the east coast of Canada with a population of just under 1 million, through an online, closed-ended, anonymous survey which was developed in consultation with a community advisory committee. Inclusion criteria consisted of self-identifying as LGBTQ and/or offering primary health care to LGBTQ patients, being able to understand English, being 16 years of age or older, and having lived in Nova Scotia for at least one year. A total of 392 participants completed the anonymous online survey, of which 283 participants self-identified as members of the LGBTQ communities and 109 self-identified as health care providers, of whom 53 self-identified as non-LGBTQ and 56 self-identified as LGBTQ. Data were managed, analyzed and descriptive statistics generated using SPSS Version 20™.

### Measures

The online survey was developed collaboratively between the research team and the community advisory board following both the completion of a scoping review on the key factors impacting LGBTQ health, as well as community consultations in urban and rural Nova Scotia with community stakeholders. The resultant survey consisted of closed-ended questions related to sociodemographic factors, self-perceived health status, and health care experiences. The health care provider survey also included a section on their experiences and perceptions regarding LGBTQ population’s access to health care and physician-patient interactions. The anonymous, online survey was completed electronically by selecting radio buttons, checking boxes, and typing in text, depending on the nature of the question.

### Procedures

The survey was posted online using Opinio following approval by the Dalhousie University Research Ethics Board. The survey remained open for a total of six months.

## Results

The key results are presented in the following sections, starting with LGBTQ respondents (*N* = 283), followed by health care providers who did not self-identify as LGBTQ (*N* = 53), and LGBTQ self-identified health care provider respondents (*N* = 56).

### LGBTQ populations’ perspective

#### Sample description

Of the respondents who completed the survey, the mean age was 32 years. Respondents self-reported sexual orientation, gender identity/expression, employment status, education, and ethnicity are offered in Table 1.

**Table 1:** Demographics

Variables	Percent
Sexual orientation^a^	
Gay	27.2
Lesbian	25.8
Bisexual	30.0
Queer	38.5
Questioning	3.5
Heterosexual	2.8
Other options (mostly pansexual and asexual)	13.8
Gender identity/expression	
Cisgender female	40.3
Cisgender male	13.3
Transgender/transsexual male to female	5.3
Transgender/transsexual female to male	9.9
Two spirit	3.2
Genderqueer/non binary (or otherwise gender variant)	18.7
Other options (mostly gender fluid or intergender)	9.3
Employment^a^	
Full-time position	41.3
Part-time position	16.3
Unemployed	12.7
Full-time student	32.5
Religion or faith	
Raised in Christianity	55.8
Raised in other faiths	10.6
Not raised in any religious beliefs	33.6
Self reported ethnicity	
White	88.0
Mixed heritage	5.3
Aboriginal	1.8
Black	1.1
Asian	0.4
Not indicated	0.4

LGBTQ sample description

^a^Sum do not come up to 100% because respondents were allowed to select all that apply

#### Self-rated health status

The majority of respondents defined both their self-perceived physical and mental health statuses to be Good/Somewhat Healthy (a score of 4 out of 5). In addition, the majority of respondents indicated that their health status has remained approximately the same in the last year, and rated their own health literacy as very good.

#### Relationship with the health care system

The majority of respondents reported having undergone a routine check-up within the last 12 months, reported having a primary health care provider or family doctor, and reported being satisfied with them. However, the majority also reported being uncertain about the level of LGBTQ-friendliness of their family doctor, their knowledge and cultural competence about LGBTQ issues, and the inclusiveness of the health care system in Nova Scotia. Both positive and negative interactions are offered in Table 2.

**Table 2:** Interactions with the health care system

		Percent
Positive interactions		
At least one good experience	LGB	66.2
with the health care system	trans	68.7
Negative interactions		
At least one bad experience	LGB	36.4
with the health care system	trans	55.5

Interactions with the health care system

#### Perceived importance of health-related topics regarding one’s own health

The key self-perceived health-related topics for LGBQ, and trans individuals are offered in Tables 3 and 4, from most to least important.

**Table 3:** Perceived importance of health related topics regarding one’s own health

Perceived importance of health related topics regarding one’s own health	
LGBQ individuals	
Reproductive health and family planning	
Sexual health	
Problematic substance use (drugs and alcohol)	
Access to harm reduction supplies (clean syringes, pipes, filters, alcohol swabs, safe sharps deposit…)	
Access to safer sex supplies (e.g. condoms, dental dams)	
HIV/AIDS	
Diabetes and obesity	
Trans individuals	
Sexual health	
Transition services for trans individuals	
Reproductive health and family planning for trans individuals	
Positive body image, self-esteem and coping strategies	
Anxiety/stress and other mental health concerns	
Access to safer sex supplies (e.g. condoms, dental dams)	
Supportive housing	
Nutrition/healthy eating	
Healthy aging

**Table 4:** Sexual orientation/gender Identity of LGBTQ health care providers

Sexual orientation^a^	Percent
Gay	32.1
Lesbian	41.1
Bisexual	12.5
Queer	32.1
Questioning	3.6
Heterosexual	1.8
Other options (“inclusive”)	1.8
Gender identity/expression	
Cisgender female	37.5
Cisgender male	23.2
Transgender/transsexual female to male	5.4
Genderqueer/non binary (or otherwise gender variant)	10.7
Not to disclose	8.9
Other options^b^	10.7
Not indicated	3.6

Sexual orientation of health care providers who identify as LGBTQ

^a^sum does not add up to 100% because respondents were allowed to select all that apply

^b^mostly “woman”, “female” and “male” (which suggests that some participants did not know the meaning of the term cisgender)

#### Pathways to health

Overall, the factors that were perceived to positively contribute to their health and well-being were similar for LGBQ and trans populations. These factors included: a) self-care, b) personal coping skills, c) self-esteem, d) safe and inclusive school or work environment, e) social support, f) access to LGBTQ-friendly/safe spaces, and g) community mental health resources.

### Non- LGBTQ self-identified health care providers’ perspective

#### Sample description

**Fig. 1:**
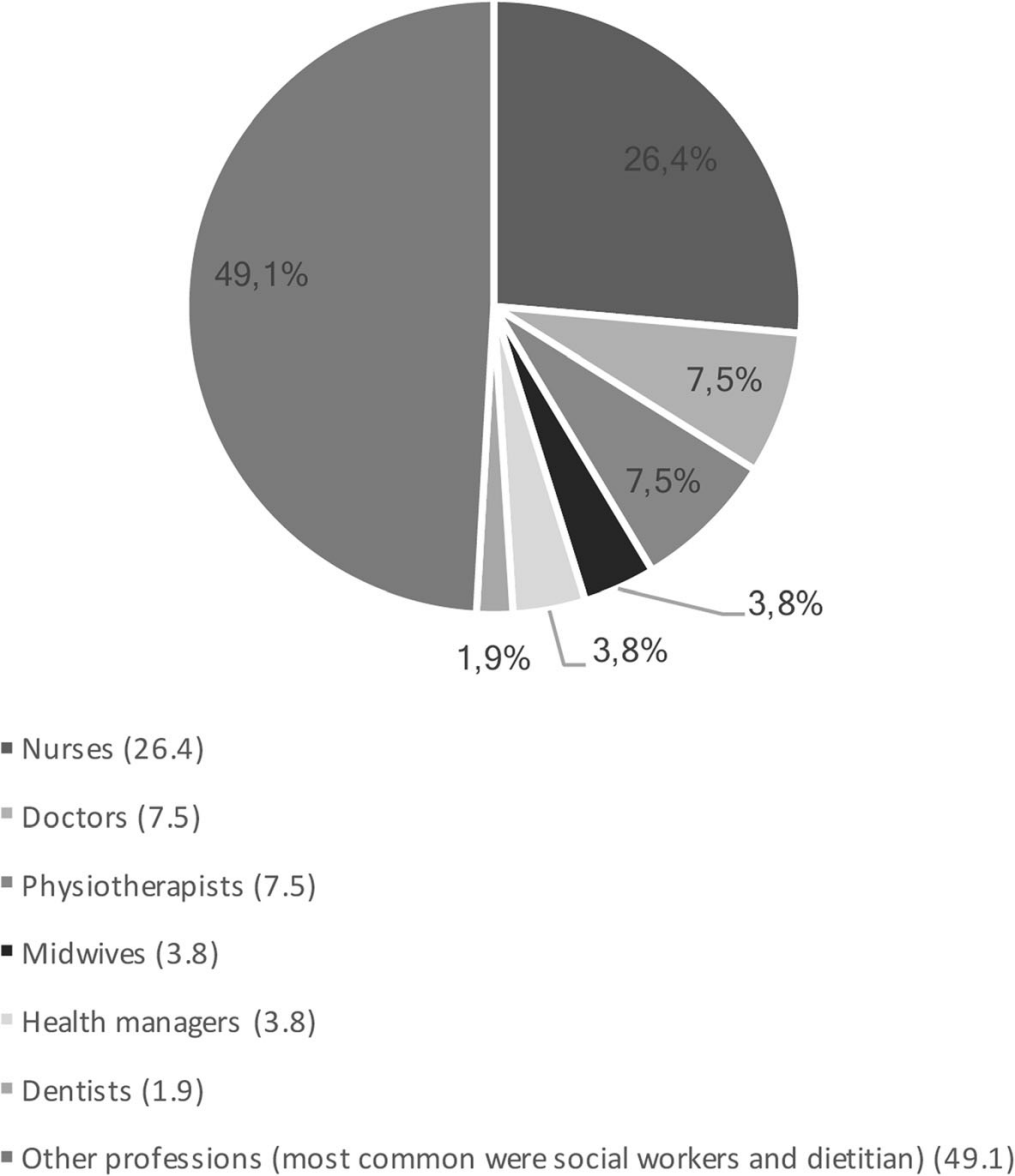
Types of health professionals (Non-LGBT self identified care providers’ perspective)

A total of 53 surveys were completed by health care providers from across Nova Scotia who did not self-identify as LGBTQ but who offer care to LGBTQ community members. In addition to answering sociodemographic questions, respondents offered their experiences and perceptions regarding LGBTQ populations’ access to health care and physician-patient interactions. Respondents’ average length of experience as a health care professional was 14 years, and types of health care professionals are offered in Fig. 1.

**Fig. 2:**
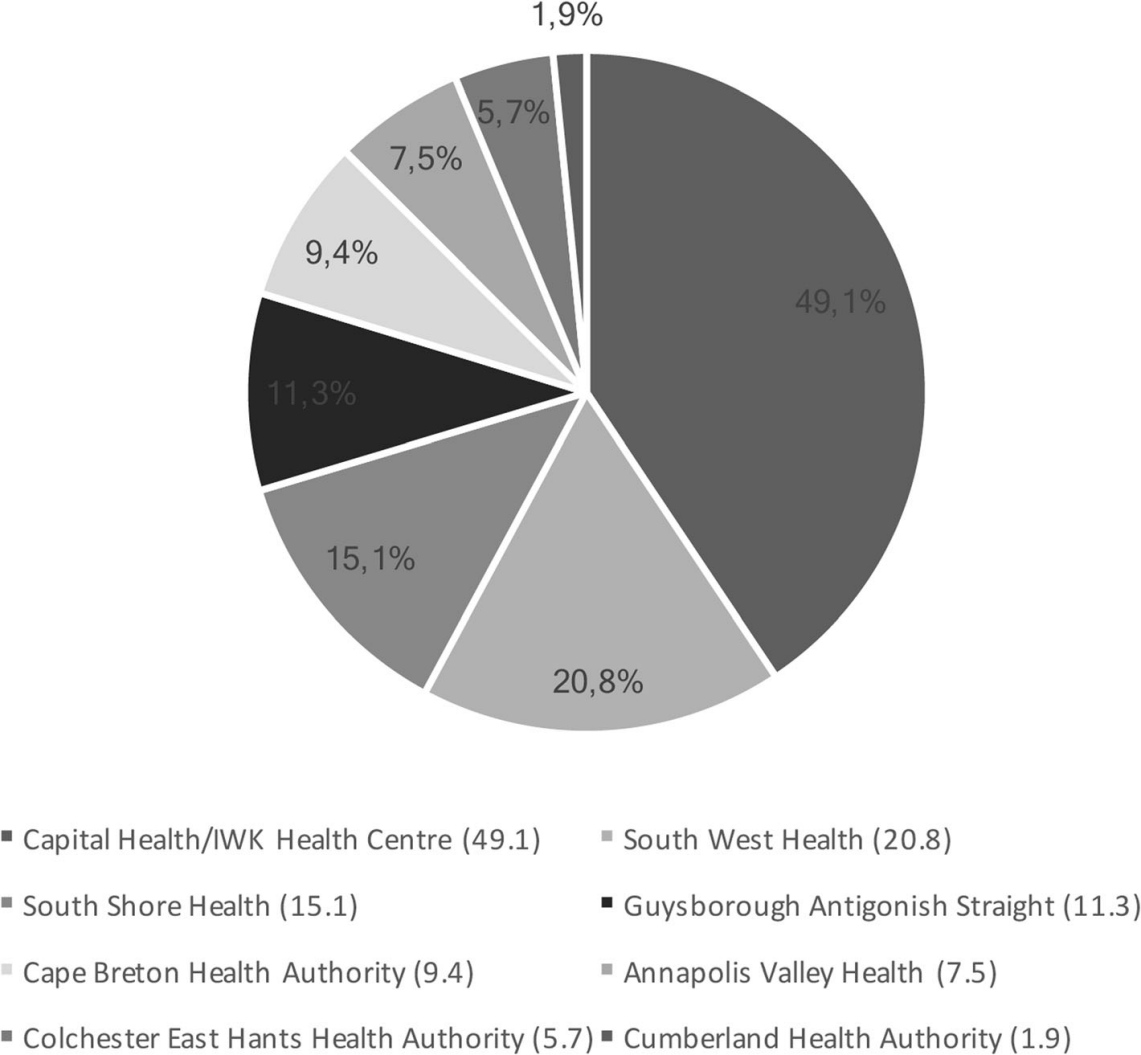
Place of work (Non-LGBT self identified care providers’ perspective)

The majority of the health care provider respondents reported working in hospitals, primary health centres and community health centres (see Fig. 2).

#### Patient-provider interaction

The majority of the health care providers who did not identify as LGBTQ reported feeling a certain degree of discomfort when having to address LGBTQ specific issues with their patients, such as access to transition services for trans patients or family planning/reproductive health specifically for LGBTQ populations. These issues also included mental health, domestic abuse and problematic drug use. Amongst those who regularly take sexual histories of patients, most do not usually screen for non-heterosexual sexual activity. When asked about the frequency by which patients openly discuss their gender identity/expression or sexual orientation, most of the health care providers indicated that this occurs ‘sometimes’.

Many (41.5%) of the health care providers who did not identify as LGBTQ believe that the health concerns of LGBTQ populations are ‘similar’ to, but not entirely the same as those found among heterosexual, cisgender populations. However, 34% stated that the health concerns of the two populations are ‘completely different’, and 13.2% think that they are ‘the same’. The remainder reported being ‘uncertain’.

#### LGBTQ knowledge and cultural competence

Only 9.4% of non-LGBTQ identified health care providers in our survey indicated that they felt ‘very knowledgeable’ about issues related to sexual orientation and sexual behavior, and 3.8% about issues related to gender identity/expression. Over half (54.7%) of respondents reported having never received training for cultural competence in relation to LGBQ populations, and 60.4% reported having never received training for cultural competence regarding trans populations. Most of those who have received such training have done so while in-service, mostly in the form of conferences or workshops. Nearly half (49.1%) reported believing that their health care environment is inclusive of LGBTQ patients, and 35.8% are uncertain about that. Over half (58.5%) reported that their working environment is inclusive of LGBTQ staff, while 34% are uncertain about this issue.

More than 50% of the health care provider respondents who did not self-identify as LGBTQ identified the need for further education regarding LGBTQ populations (e.g. CME LGBTQ knowledge, communication skills, etc.) as very important to enhance health care services. In addition, 43.4% considered inclusive signs and posters to be very important, and 49.1% considered the language used in medical intake forms to very important.

#### Perceived importance of health-related topics in LGBTQ health

The health-related topics perceived as most important by non-LGBTQ identified health care providers were: a) sexual health, b) reproductive health and family planning, c) transition services for trans individuals, d) anxiety/stress and other mental health issues, and e) HIV/AIDS.

### Health care providers who identify as LGBTQ

#### Sample description

**Fig. 3:**
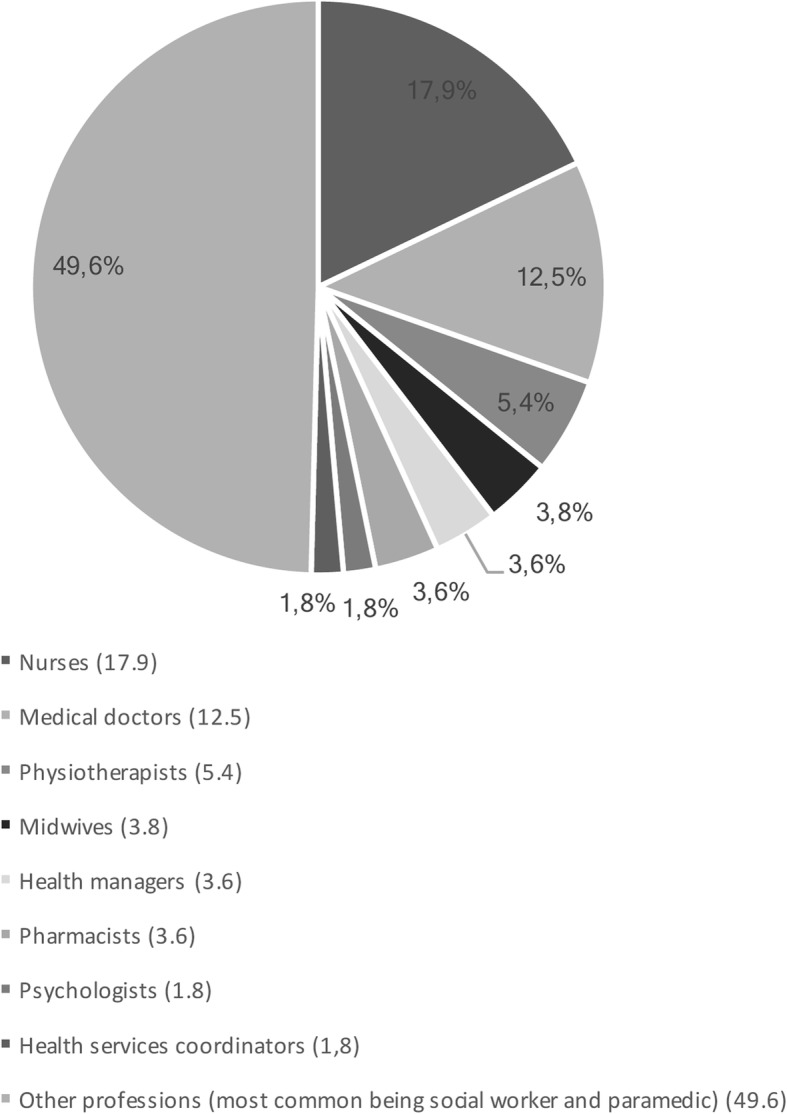
Types of health professionals (Health care providers who identify as LGBTQ)

We obtained a total of 56 completed surveys from health care providers in Nova Scotia who self-identify as L, G, B, T or Q (LGBTQ). The mean age of these participants was 41 years, and their sexual orientation and gender identity/expression are offered in Respondents’ average duration of experience as a health care professional was nine years, and their professional training is offered in Fig. 3.

**Fig. 4:**
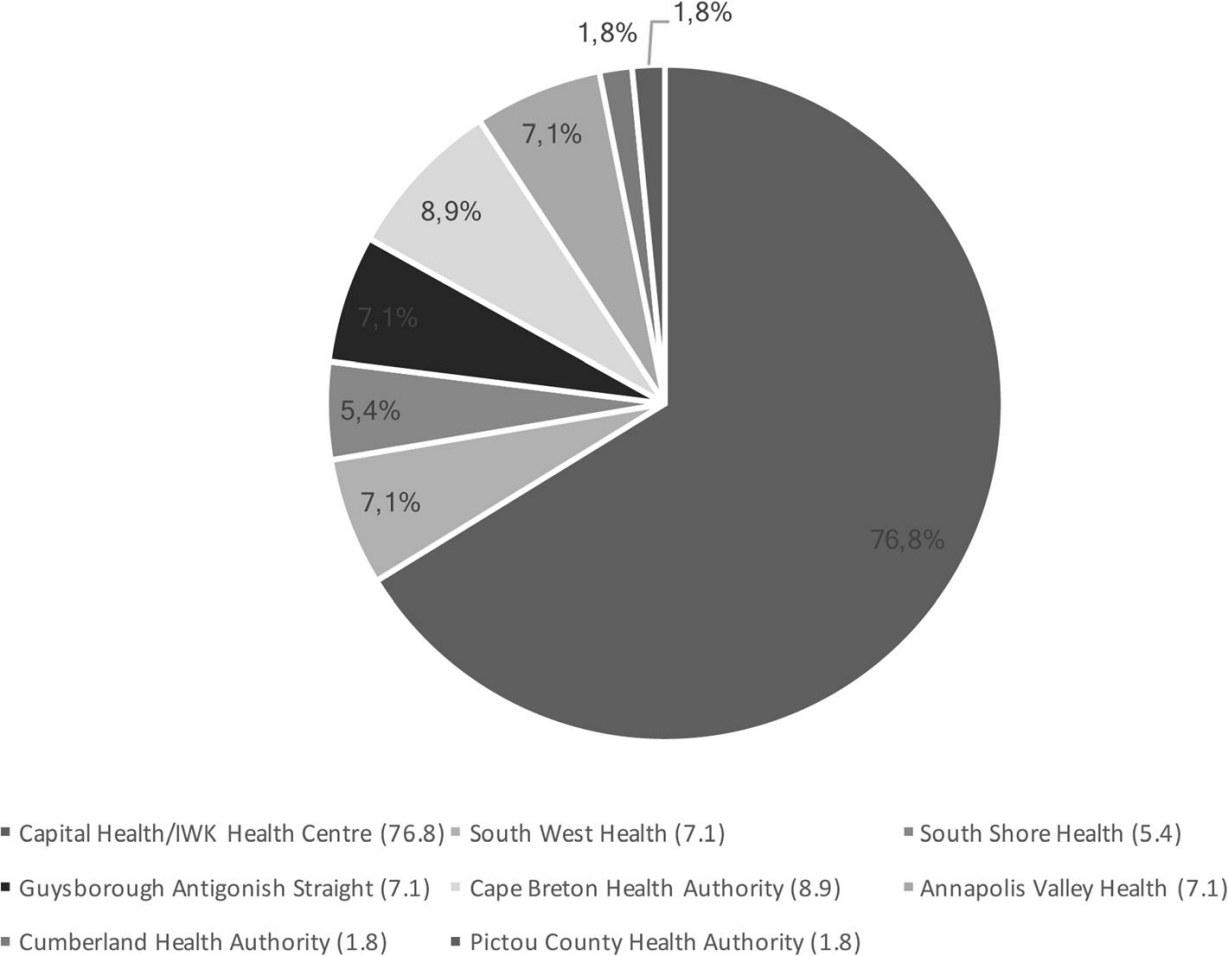
Place of work (Health care providers who identify as LGBTQ)

Most health care respondents reported working in hospitals, community health centres and other health services (Fig. 4).

#### Patient-provider interaction

Most LGBTQ-identified health care providers reported feeling comfortable when having to address LGBTQ specific issues with their patients, with the exception of reproductive concerns for transgender patients, where there was wide variability in levels of comfort reported. These respondents also reported feeling comfortable when addressing issues concerning mental health, domestic abuse and problematic drug use. Amongst those who regularly take sexual histories of patients, most reported always or often screening for non-heterosexual activity. However, no differences were observed regarding the reported frequency with which patients openly discussed their gender identity/expression or sexual orientation with health care professionals.

Unlike what was observed among the non-LGBTQ identified health care provider respondents, the majority (48.2%) of LGBTQ-identifying health care providers agreed that the health concerns of LGBTQ populations are different than the ones of heterosexual, gender-binary populations. Specifically, 33.9% stated that they are similar, only 3.6% think that they are the same, and 8.9% reported being uncertain of what to think about this question.

#### LGBTQ knowledge and cultural competence

Over one quarter (28.6%) of the LGBTQ-identified health care providers reported feeling very knowledgeable about issues related to sexual orientation and sexual behaviour, as opposed to 9.4% of those in the general group (non-LGBTQ), and 50% feel somewhat knowledgeable. In addition, 17.9% reported feeling very knowledgeable about issues related to gender identity/expression, versus 3.8% of the general group, and 42.9% reported feeling somewhat knowledgeable. The perception of not being knowledgeable enough is a more common response regarding trans patients than LGBQ patients. Just over half (53.6%) of the sample reported not having received any training for cultural competence about LGBQ populations. In the same way, 67.9% had never received training for cultural competence about trans populations. Again, most of those who have been trained have received this education while in-service. Further, 46.4% of health care professionals reported that their health care environment is inclusive of LGBTQ patients, slightly less than their heterosexual counterparts; and 23.2% are uncertain about that, which means that professionals identifying as LGBTQ have more solid views on whether the health system is inclusive or not. Nearly 60% (58.9%) believed their working environment was inclusive of LGBTQ staff, while 26.8% remain uncertain; however, only 10% reported that their working environment was definitely not LGBTQ inclusive.

Compared to the group analyzed in the previous section, health care providers considered the need for non-heterosexist medical intake forms as more important (55%). Some professionals expressed the need for specific measures, such as gender-neutral bathrooms in health centers.

#### Perceived importance of health-related topics in LGBTQ health

The health-related topics perceived as most important by LGBTQ-identifying health care providers were: a) anxiety/stress, b) depression, self-harm and suicidal ideation, c) positive body image, self-esteem and coping strategies, d) sexual health, and e) transition services for trans individuals.

In general, both subgroups of healthcare providers (LGBTQ-identifying and non) considered obesity, cancer screening and cardiovascular health amongst the least important health-related issues relative to others, despite the fact that approximately 40% of them consistently rate these topics as very important.

### Limitations

It should be noted that closed-ended online surveys do have limitations, particularly where the questions are based on self-perceptions and hence subject to variable interpretation of the concepts used.

## Discussion

The results of this online survey help bring to light a number of key issues related to improving pathways to LGBTQ primary health in Nova Scotia from the perspectives of both LGBTQ community members and health care providers. Specifically, it is noteworthy that more than one third of LGBQ respondents and more than half of trans respondents have had at least one poor experience with the health care system. Despite this, LGBTQ respondents rated their mental and physical health status as good overall, and most reported having a primary health care professional who they believe they can turn to for health matters.

In moving forward, it is important to note that there are key differences in the health-related topics perceived as most important for LGBQ versus trans individuals. Overall, these include reproductive health and family planning, sexual health, problematic substance use (e.g. drugs and alcohol), and access to harm reduction and safer sex supplies. In addition, trans individuals are also concerned with transition services, body image, self-esteem and coping strategies, and mental health issues, such as anxiety/stress, depression, or self-harm. Supportive housing, nutrition/healthy eating and healthy aging are also perceived as important for trans individuals.

Factors that were seen to positively contribute to overall health and wellness are very similar for LGBQ and trans populations and include self-care, personal coping skills, self-esteem, a safe and inclusive school or work environment, social support, access to LGBTQ-friendly/safe spaces, and community mental health resources. Given that health concerns and priorities typically vary along the life course [16] it is important to view our study results from a life course perspective, which recognizes that social, cultural, and economic contexts shape experiences of health [22]. Many of the health issues highlighted in our survey represent a life course approach in understanding for example, issues related to sexually transmitted diseases, drug-consumption habits, dietary patterns, or pregnancy/fertility which are typically more relevant for people at early stages of adulthood, while the majority of cancers and cardiovascular or metabolic diseases typically present in later stages. Clinical guidelines recommend screening practices on the basis of risk, which implies that such practices are usually targeted towards particular subpopulations in which their effect can be more beneficial. The relatively young mean age of our sample may have had an influence on the perceived importance given by participants to different health-related topics.

From a health care providers’ perspective, it is noteworthy that the perceived health related concerns of their LGBTQ patients do not always fully coincide with those expressed by LGBTQ individuals themselves. Specifically, many health care providers do not feel knowledgeable, comfortable or culturally competent enough when it comes to LGBTQ populations and their primary health care needs and this may be related to the lack of formal training on these issues in their medical education or in available Continuing Medical Education (CME) [23] training. There are differences between the comfort, perceptions of knowledge, and perceptions of LGBTQ inclusiveness of health services – both for patients and for professionals – between health care providers, regardless of their sexual orientation. However, even those identifying as LGBTQ expressed the need for additional training.

## Conclusion

In general, respondents pointed to a variety of health-related issues that are of an intersectional and health equity nature [24, 25]. An intersectional and health equity approach points out the importance of the overlapping and intersecting axes of oppression such as gender, race, age, class and sexuality [26, 27].Our findings suggest that pathways to primary health for LGBTQ populations may be improved by addressing issues at the micro level of the individual (e.g. additional training and awareness) as well as at the macro systems level (e.g. health care systems, processes and procedures). Additional research in meeting the unique primary health needs of LGBTQ populations and health care providers is warranted, particularly given the complex and evolving interplay between micro and marco level factors that can collectively and synergistically impact on health outcomes among more socially marginalized populations. Our findings highlight the need to continue working towards the development of a truly welcoming, health equity focused, inclusive and culturally competent primary health system for all Nova Scotians, including LGBTQ populations.
